# Profuse Nocturnal Enuresis

**Published:** 1893-08

**Authors:** T. F. Allen


Profuse Nocturnal Enuresis.—A boy of five years was passing frequently large quantities of pale urine; every night, besides being taken up two or three times to urinate, he soaked two mattresses.

His stools are of normal consistence and regular, but gray, or mixed gray, in color. He is irritable and seems puffy under the eyes. He eats heartily and sleeps soundly. *Plantago*$^{200}$, (Jenichen.)

Three doses seemed to restore a normal condition, his urine became natural in quantity, and he no longer wet his bed at night, his stools became natural in color, and he was no longer irritable.

T. F. Allen, M. D., *in Journal of Mat. Medica.*

